# Assessing the effects of conductivity on egg development and survival of Eastern Hellbenders (*Cryptobranchus a. alleganiensis*)

**DOI:** 10.1038/s41598-024-82969-5

**Published:** 2025-02-25

**Authors:** Ryan K. Brown, Matthew D. D. Kaunert, Kelly S. Johnson, Willem M. Roosenburg, Viorel D. Popescu

**Affiliations:** 1 Department of Biological Sciences, Ohio University, Athens, OH USA; 2 Clean Water Institute, Lycoming College, Williamsport, PA USA; 3 Department of Ecology, Evolution and Environmental Biology (E3B), Columbia University, New York, USA; 4 Center for Environmental Research, University of Bucharest, Bucharest, Romania

**Keywords:** Ecology, Environmental sciences, Herpetology

## Abstract

Increasing concentrations of dissolved ions in freshwater ecosystems often stem from anthropogenic sources and have been implicated in the decline of sensitive aquatic organisms. Eastern hellbenders (*Cryptobranchus a. alleganiensis*) are fully aquatic salamanders that are experiencing range-wide declines and increased conductivity has been suggested as a cause. Declining populations are skewed towards older age classes, indicating a lack of successful reproduction (i.e., population recruitment). Therefore, insights into mechanisms that may cause mortality in early life stages are of great value to hellbender conservation. We conducted an experimental study to evaluate the effects of increased conductivity on the survival and development of hellbender eggs and newly hatched larvae. Wild-collected eggs were incubated across a range of conductivities (100, 300, 600, 1,000 µS/cm). We used two types of salt, aquarium salt and rock salt, to manipulate conductivity during and after hatching. Overall mortality rates were low (< 0.07) across all treatments, but highest in the 1,000 µS/cm treatments (0.14). There was no difference in mortality between aquarium salt and rock salt treatments, however the rock salt treatment stimulated earlier hatching times. Larvae reared in the 1,000 µS/cm treatments had shorter snout-vent length (SVLs) (mean = 25.44 mm) than those reared at 300 µS/cm (mean = 26.95 mm) and marginally smaller head and mid-section width than individuals reared in the 100 µS/cm treatments. Our study suggests that higher conductivity has limited effects on egg and larval survival and development, and that water conductivity alone may be a suboptimal metric relating the persistence of hellbender populations to water quality. In addition, the type of salt affected hatching rates and timing, thus evaluating the composition and concentration of various ions leading to elevated conductivity is essential to understanding how degraded water quality may affect early life stages of this imperiled amphibian.

**Supplementary Information:**

The online version contains supplementary material available at 10.1038/s41598-024-82969-5.

## Introduction

Freshwater ecosystems are some of the most imperiled ecosystems in the world, with higher declines in biodiversity than most terrestrial systems^1^. Freshwater ecosystems are uniquely sensitive to human land use practices due to their connectivity to other parts of the landscape, acting as ‘receivers’ of anthropogenic pollutants^2^. In the United States, there is a long history of stream alteration due to land use change, such as agriculture, mining, and urban development, as well as habitat degradation via dams and channelization efforts, leading to precipitous declines of sensitive freshwater species^3^. While toxic substances introduced from these land use practices such as pesticides, heavy metals and fertilizers are known to negatively affect aquatic life, alterations made to the physical and chemical properties of water can also be deleterious. For example, lower pH associated with processes such as acid rain or acid mine drainage leads to extirpation of aquatic invertebrate and vertebrate fauna^4–7^.

Another potential concern is excess concentrations of dissolved ions, which correlate with increased sedimentation and intensifying land use practices^8–10^. While some streams have naturally higher ionic concentrations due to local geology, many streams in regions of the eastern United States, such as Appalachia, have naturally low ion loads^11,12^. The total concentration of ions in freshwater systems has been increasing in many areas of the United States, primarily due to increased anthropogenic activities^13^. Sources of dissolved ions include weathering of concrete infrastructure in urban areas, effluents from crude oil and natural gas exploration and production, agricultural runoff, application of deicing salts, and industrial processes^12,14,15^. Electrical conductivity is a measurement of dissolved ion concentrations and is elevated, sometimes over 10-fold, in many streams of the eastern US where historical conductivities were often < 100 µS/cm (microSiemens per cm)^9,11,16^. Elevated conductivities of specific ion concentrations have been shown to negatively affect an array of freshwater biota including bacteria, invertebrates, fish, reptiles, amphibians, and plants^14,17^. Amphibians actively transport ions across the skin membrane to maintain osmotic balance, making them particularly sensitive to changes in their physiochemical environment, and studies have found that exposure to elevated ion concentrations can negatively affect amphibian behavior, growth, and survival^17–20^. In this study, we investigated the effects of elevated ion concentrations on egg development, hatching and larval survival in an imperiled stream amphibian, the Eastern hellbender (*Cryptobranchus a. alleganiensis*).

The Eastern hellbender is a fully aquatic giant salamander native to streams in parts of the eastern United States. Hellbenders are an indicator species of stream health, requiring clean, flowing, highly oxygenated water^21^. Hellbender populations are exhibiting rapid declines across much of their range, the causes of which are not fully understood, but sedimentation, habitat loss and degradation, endocrine disruption, pollution, disease, species introductions and collection are thought to be possible factors^22,23^. Many remaining populations of hellbenders are skewed towards larger adults, suggesting that low recruitment (i.e., lack of successful reproduction) is contributing to these declines^23,24^. One of the largest gaps in our understanding of hellbender life history is survival data for the eggs and larvae. Elevated conductivity has been suggested to affect egg and larval survival thereby reducing recruitment but this has never been tested empirically. Several conductivity thresholds have been associated with hellbender absence^10,25,26^, Pitt et al. (2017)^26^ reported that hellbenders were absent from streams where conductivity exceeded 278 µS/cm in the Susquehanna River watershed; Da Silva Neto et al., (2021)^27^ postulated that hellbenders may show declines when conductivity was > 50 µS/cm.

The goal of this study was to assess the effects of elevated conductivity levels on the survival and development of hellbender early life stages (eggs and larvae). Specifically, our objectives were: (1) to quantify the effects of various conductivity levels (100–1000 µS/cm) on egg and larval survival and development, (2) to evaluate sublethal effects on elevated conductivity on larval morphology. We conducted a controlled experiment in which we manipulated water quality using two types of salt (aquarium salt and rock salt) and assessed survival, hatching success, and post-experiment morphometric differences between surviving larvae raised in each conductivity level. We predicted that high conductivity (600, 1000 µS/cm) would result in higher egg and larval mortality and reduced or delayed hatching rates. We hypothesized that higher physiological stress induced by high conductivity would have sublethal effects such as slower development rates. We also hypothesized that rock salt would result in lower egg and larval survival compared to aquarium salt treatments due to the larger mixture of ions present in the aquarium salt. Overall, our study is the first to empirically evaluate the effects of elevated conductivity on survival of hellbender early life stages, and thus fills an important gap in our understanding of the persistence of one of the most imperiled amphibians in North America.

## Methods

### Egg collection

**Fig. 1:**
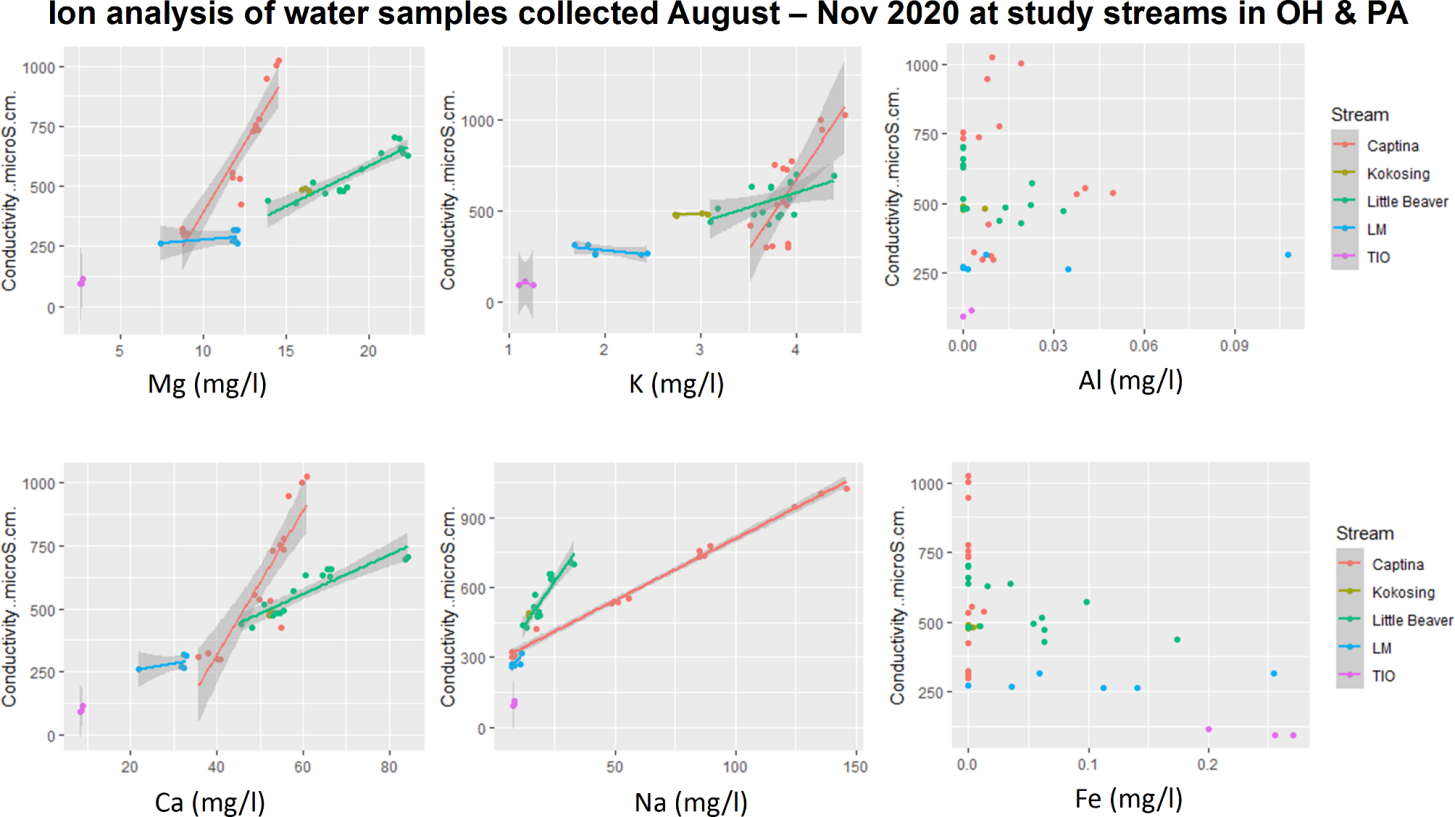
Select cation concentrations and conductivity ranges of occupied hellbender streams, recorded during August – November 2020; this period of time is the typical hellbender breeding season in OH (Captina Creek, Little Beaver Creek, Kokosing) and PA (Tionesta Creek (TIO), Little Mahoning Creek (LM)), with the onset of breeding in August, egg deposition in early September and egg hatching in October.

We collected hellbender eggs from two streams (Tionesta Creek and Little Mahoning Creek) in western Pennsylvania, USA on 20 and 21 September 2021. Little Mahoning Creek eggs (*N* = 80 eggs) were collected on 20 September 2021 from two nests deposited in artificial hellbender nesting structures (hereafter nest boxes) deployed as part of a research project investigating reproductive ecology of hellbenders^28^. The two streams have relatively low conductivity (Fig. 1) relative to the range of experimental conductivity levels in this study (Tionesta = 100 plus minus, Little Mahoning − 200 plus minus), based on a separate ion load study conducted between August and November 2020 at several artificial nest box deployment sites in Ohio and Pennsylvania streams^28^. We located nests in Tionesta Creek by following adult males tagged with VHF transmitters, part of a short-term pre-reproduction telemetry study^29^. We identified den sites by inserting a Depstech 1080P dual lens endoscope (Depstech, Shenzhen, China) or an AquaVu Micro Revolution Pro 5.0 underwater camera (Aqua-Vu, Crosslake MN, USA) underneath occupied shelter rocks. Four hellbender nests were located under natural shelter rocks in Tionesta. Eggs were collected using a piece of metal wire bent into a small hook-shape on the end and coated with electrical tape to reduce harm to the guarding male hellbender and eggs. A total of 320 eggs were collected from the four nests in Tionesta (*N* = 80 eggs per nest from two nests, *N* = 120 eggs from one large nest and *N* = 40 eggs from one small nest). All collected eggs were transported to the Ohio University Athens campus immediately after collection using 19-quart Engel live bait coolers (Engel Coolers, Jupiter FL) fixed with battery powered air pumps; we placed unique clutches in individual mesh bags to keep them separated. Survival of all transported clutches from field sites to the lab (~ 6 h transportation time) was 100%. All eggs were fertilized and developing at the onset of the experiment. Permission for egg collection was provided by the Pennsylvania Fish and Boat Commission (permit # 2021-01-0192).

### Experimental setup

We conducted the experiment in a temperature-controlled environmental chamber on the Ohio University Athens campus. The temperature of the room was set to 17 °C to mimic average water temperatures in natal streams at the time eggs were collected. Forty 5-gallon (19-liter) tanks were set up, each housing 10 hellbender eggs from the same clutch. The tanks were arranged on two shelving units each containing four shelves and five tanks per shelf. Each tank was fitted with an “egg cradle” made from Tenax ™ black plastic extruded mesh fencing (Mesh Size 3/4-in x 1-in) and ½ inch PVC pipe. The egg cradles held the eggs over a 4-inch air stone which was placed underneath the lowest point in the middle of the cradle where the eggs would naturally settle. The air stones were powered by two Pond Master™ air pumps (model AP-40) (Danner Manufacturing, Islandia NY) that were attached to a network of ½ inch PVC pipe fitted with Aquaneat Aquarium™ 3/16-inch 1-way thread metal gang valves (one per tank). Each pump powered 20 air stones (one air pump per shelf). The pumps were set on a timer to turn on for 30 s and then turn off for 45 s. This process mimics natural paternal care documented by hellbenders, where eggs are periodically agitated or tail-fanned by guardian males. This agitation has been shown to be required to prevent adhesion of hellbender embryos to the egg membrane which causes mortality (N. Burgmeier, pers. comm.).

### Conductivity manipulation and egg monitoring

We exposed hellbender eggs to four conductivity levels (100, 300, 600 and 1000 µS/cm) (Table 1) that were maintained in experimental tanks using two types of salt type treatments: Flora Viv Reconstitute RO™ mineral element powder (hereafter aquarium salt) (Continuum Aquatics, Fort Payne AL), a type of salt that is commonly used in aquaria to maintain a balanced ion composition that is conducive to aquatic life, and rock salt (99.6% pure sodium chloride Diamond Crystal™ “Solar Naturals Salt Crystals”). We used commercial aquarium salts because they are a balanced mixture of ions suitable for aquatic life in captive conditions; we used rock salt because sodium (Na+) and chlorine (Cl-) ions specifically, are known to enter aquatic systems from road deicing salts, effluent from natural gas production, and agricultural runoff 32. In addition, preliminary water sampling showed Na + to be the dominant cation in hellbender-occupied streams exceeding 1,000 µS/cm^28^. We selected the range of 100–1000 µS/cm because preliminary water samples collected across several watersheds in Ohio and Pennsylvania showed that hellbenders persisted in streams within this range of conductivity (Fig. 1, see Ion Content Analysis section of Methods).

**Table 1:** Average water quality parameters for each conductivity level and treatment type.

Conductivity Level (µS/cm)	Treatment	Mean conductivity (µS/cm)	pH	DO (mg/L)	Temp (°C)	GH	KH	NO_2−_	NO_3−_
100	Aq. Salt	114.12 ± 0.1	7.05	8.67	15.49	15	0	0	0
300	Aq. Salt	302.79 ± 0.14	7.07	8.67	15.76	15	10	0	0
600	Aq. Salt	571.42 ± 0.28	7.14	8.71	15.54	22.5	30	0	0
1000	Aq. Salt	950.41 ± 0.34	7.12	8.54	15.52	30	30	0	0
100	Rock Salt	111.52 ± 0.11	7.02	8.60	15.56	15	0	0	0
300	Rock Salt	303.70 ± 0.19	7.02	8.65	15.59	15	5	0	0
600	Rock Salt	577.14 ± 0.32	7.04	8.57	15.67	15	0	0	0
1000	Rock Salt	959.33 ± 0.4	7.05	8.63	15.65	15	16.7	0	0

Values of general hardness (GH) were similar between treatments while carbonate hardness (KH) was higher in the aquarium salt treatment. Levels of nitrate or nitrite in tanks were below the detection limit of the tests.

All tanks were initially filled with reverse osmosis (RO) water with added aquarium salt to reach a base conductivity level of 100 µS/cm. Each salt treatment was assigned to one shelf, and conductivity levels were randomized for tanks within a shelf. The rock salt was dissolved in water to its saturation point and then was added to the 100 µS/cm control water in concentrations to achieve the desired conductivity levels. Eggs from the six unique clutches (10 per tank from a given clutch) were divided evenly between the salt and conductivity treatments, with duplicates of the large *N* = 120 egg clutch being used in the rock salt treatment (80/120 eggs) and the small *N* = 40 egg clutch only being used in the aquarium salt treatment.

We measured conductivity levels weekly using a YSI Professional Series Pro 2030 Dual Dissolved Oxygen/Conductivity Instrument (Yellow Springs Instruments, Yellow Springs, OH) to ensure that the desired levels remained constant, as well as pH, dissolved oxygen, and temperature (Table 1). Other water quality parameters such as general and carbonate hardness and nitrate and nitrite levels were also recorded periodically using API 5 in 1 Aquarium Test Strips™ (Mars Inc., Chicago, IL) (Table 1). As water evaporated from the tanks, conductivity tended to increase slightly, and we adjusted by adding small amounts of control water (100 µS/cm) to tanks. Eggs/embryos were monitored daily to check for (1) hatching, and (2) embryo or larval mortality. An egg was determined to be ‘hatched’ when the embryo emerged from the gelatinous egg sac and was free-swimming. At this stage we defined embryos as larvae. Embryo mortality was determined by observing egg condition. Shortly after expiration, embryonic yolk-sacs would begin to disintegrate and the eggs became cloudy and opaque. Dead eggs were removed from the tanks. The eggs and larvae were monitored from 21 September to 1 December 2021, when we ended the experiment; this period reflected the hellbender natural time to hatching and approximately the first month of the larval stage. Hellbender larvae do not require food at this stage of life because they sustain themselves on their yolk-sac for several months post-hatching^30^ (S. Royal, Indianapolis Zoo, pers. comm).

### Statistical analysis

We used a binomial generalized linear mixed model with a logit link to assess the difference in mortality between salt and conductivity treatments at the end of the experiment using R package *lme4*^31^. We used the proportion of larvae alive per tank (i.e., survival per tank) at the end of the experiment as the response variable. Larvae were considered to be ‘alive’ even if they did not hatch by the deadline but were alive inside the egg sac. We first tested an interaction model of conductivity × salt treatments and evaluated the contribution of each treatment type and the interaction term using an ANOVA (function *Anova* in R package *car*)^32^. If significant, we evaluated the differences in survival between conductivity treatments using pairwise comparisons in R package *lsmeans*^33^, using a Holm correction for multiple comparisons. We conducted a pairwise analysis because it directly relates to our question about the biological relevance of conductivity thresholds for persistence proposed in the literature. We also used conductivity levels as a continuous variable and ran a similar binomial model to illustrate the linear relationship between survival rate and conductivity. Lastly, we estimated time to hatching between the salt and conductivity treatments.

### Morphological measurements

At the end of the experiment all surviving larvae were photographed in a separate tank with 1 cm of water and laid over a 1-cm grid paper. At this time all larvae that remained encapsulated in egg sacs were manually hatched by making a small incision in the egg membrane; the hatched and unhatched larvae were photographed separately. We then used software ImageJ™ to measure snout-vent length (SVL), total length, mid-section width, head width and tail width of all surviving larvae.

Differences in larval morphology between conductivity and salt treatments were evaluated using MANOVA (across all five measurements) and linear mixed effects models (using R package *lme4*)^31^ using the interaction model conductivity × salt treatments. Separate models were run for each morphometric measurement as the predicted variable.We tested several random effects - random intercepts for tank and clutch [1|tank], [1|clutch] and [1|tank + 1|clutch] - using a similar parameterization for fixed effects. We evaluated models using AICc and selected different random intercepts for *tank* and *clutch* separately based on the lowest AICc score. We evaluated the contribution of each treatment type and the interaction term for each of the five measurements using an ANOVA (function *Anova* in R package *car*)^32^. If the treatment type or interaction term was significant, we evaluated the differences in survival between conductivity treatments using pairwise comparisons in R package *lsmeans*^33^, using a Holm correction for multiple comparisons. The multivariate analysis (MANOVA) did not yield new information relative to the individual metrics models and we decided to report the individual metric model results as they are easier to interpret.

### Water ion content analysis

We collected water samples from each tank during the experiment on 6 October 2021, when the first larvae began to hatch. Unfiltered water samples were stored in 50 ml glass bottles, and 1 mL of HNO_3_ (65% concentration by volume) was added as a preservative. Samples were sent to the Soil, Water and Environmental Lab (SWEL) at Ohio State University to quantify cation (Ca^2+^, Mg^2+^, K^+^, Na^+^, Al^3+^ and Fe^2+^) concentrations via inductively coupled plasma – optical emission spectrometry (ICP-OES). The same sampling procedures, laboratory, and analytical methods were used to evaluate within and among stream spatial variation in the ion content of stream water during and after the breeding season (August – November 2020) in Ohio and Pennsylvania streams (Fig. 1). Stream water was collected from reaches characterized as ‘runs’, which are the most suitable habitat for eastern hellbenders in our study area during normal stream flows (we avoided periods with high flows associated with heavy rain events). The water sampling protocol was similar to the lab protocol, but we collected both unfiltered and filtered water samples (using a 47-micron Whatman™ filter, Millipore Sigma, Darmstadt, Germany). We evaluated differences in concentrations of specific ions between salt and conductivity levels (conductivity × salt treatment) using ANOVA.

We also collected and tested fluid samples from inside egg sacs to determine whether there were differences in ion concentrations inside of the egg membrane. A single individual was used from each tank and eggs from same-treatment tanks were combined (5 eggs per conductivity × salt treatment) for a total of 8 total samples. Each egg sac contained ~ 1 mL of fluid, thus we had ~ 5 mL in each sample. The fluid from inside sacs was collected by cutting a small hole in the membrane and releasing the fluid into a sterile 50 mL plastic container. We tested the difference in ion content between the tank water and egg sac fluid using a Kruskal-Wallis test. Due to small volumes of the samples, we were unable to measure the conductivity of the inner egg fluid samples. All larvae survived the procedure and were released back in their respective tank. Experimental methods using animal subjects were approved and carried out under Ohio University IACUC protocol 18-L-007. All methods were carried out in accordance with relevant guidelines and regulations and are reported in accordance with ARRIVE guidelines.

## Results

### Egg and larval survival

There was no substantial mortality of hellbender eggs or larvae in any of the conductivity levels or salt treatments and > 0.93 of individuals survived to the end of the experiment. Despite the overall low mortality rates of < 0.14, conductivity level had an effect on mortality (*X*^*2*^_*3*_ = 10.77, *p* = 0.013), while the salt treatment had no effect on mortality (*X*^*2*^_*1*_ = 0.82, *p* = 0.366) and there was no interaction between tank conductivity level and salt treatment (*X*^*2*^_*3*_ = 1.77, *p* = 0.622). Mortality was slightly higher in the aquarium salt (16 dead individuals) compared to the rock salt treatment (11 dead individuals, Table 2).

**Table 2:** Mortality of hellbender eggs and larvae for each conductivity level, aggregated across salt treatments.

Conductivity Level	Starting Total Alive	No. Aq. Salt Dead	No. Rock Salt Dead	Total Dead	Mean Mortality	SD
100 µS/cm	100	1	2	3	0.03	0.483
300 µS/cm	100	1	1	2	0.02	0.422
600 µS/cm	100	6	2	8	0.08	0.789
1000 µS/cm	100	8	6	14	0.14	1.174
Total	400	16	11	27	0.0675	0.888

**Fig. 2:**
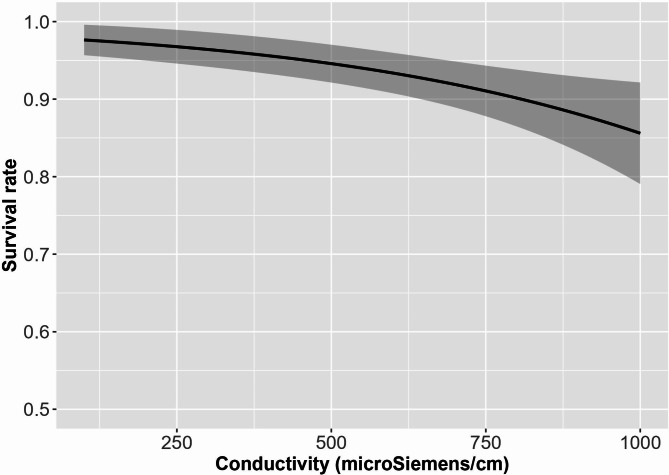
General linear model predictions for mortality using conductivity value as a numeric variable.

**Fig. 3:**
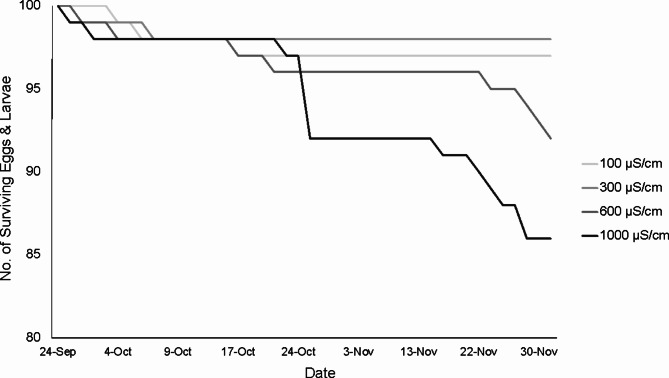
(**a**) Hellbender mortality during the experiment. Mortality increased in the final two weeks of the experiment. (**b**) Initial hatching rates of eggs raised in aquarium salt and rock salt treatments showing hellbenders reared in rock salts hatched 11 days earlier than aquarium salt treatments.

Pairwise comparisons showed a significant difference in mortality between the 300 µS/cm conductivity level and the 1,000 µS/cm level and a nearly significant difference between 100 and 1,000 µS/cm levels (Table S1). The linear relationship derived from model predictions that used conductivity value as a numeric, rather than a categorical variable, further lends support to these findings (Fig. 2). Mortality occurred mostly in the two weeks before the end of experiment when larvae were fully developed but still in egg sacs (Fig. 3a).

**Fig. 4:**
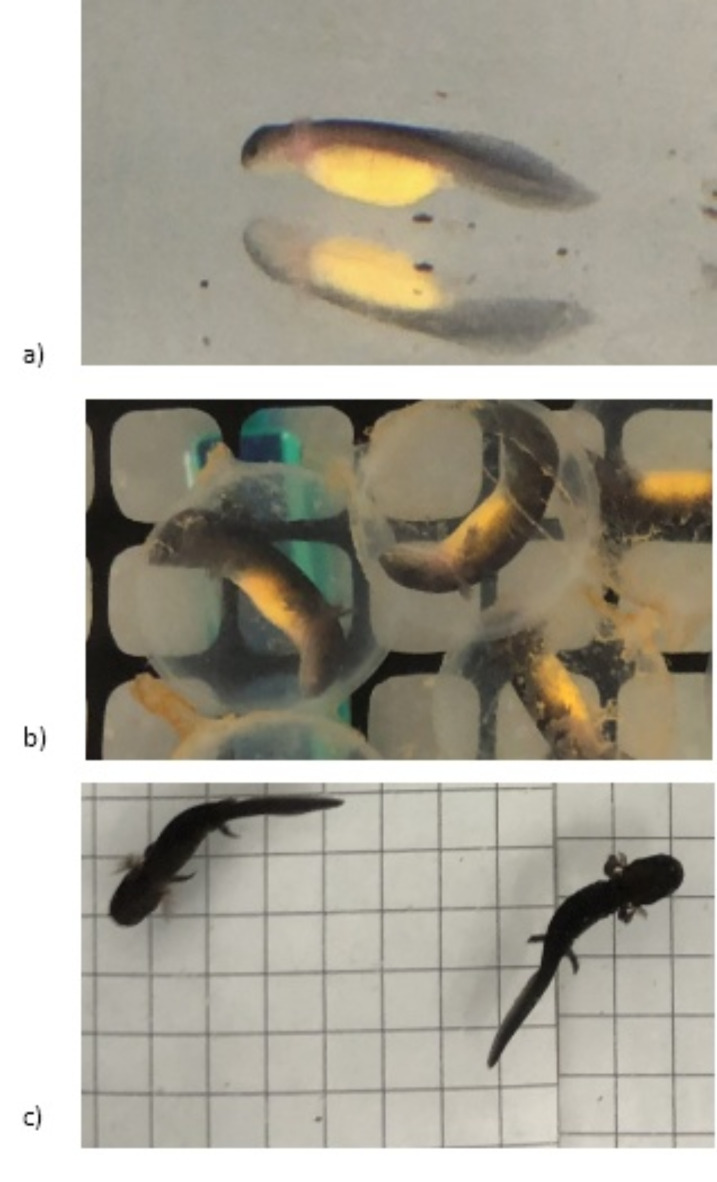
Photographs showing differentiating stages of hellbender development. (**a**) Early hatched larvae on 13 October 2021; note the lack of developed appendages. (**b**) Larvae developed enough to survive outside of egg, but still enclosed in egg sacs on 4 November 2021. (**c**) Fully developed larvae on 1 December 2021.

Hatching rates and timing yielded unexpected results. First, larvae across all conductivity levels in the rock salt treatment started hatching 11 days earlier than the larvae in the aquarium salt treatment (Figs. 3b and 4). Overall, only 67% of the larvae hatched by the end of the experiment (*N* = 71 days); the unhatched larvae were alive but appeared to not be able to break out of the egg sac. We observed a long period of hatching that lasted from the first emergence on 6 October 2021 until the end of the experiment on 1 December 2021. Overall, fewer animals hatched in the aquarium salt treatments (*N* = 116 individuals) compared to the rock salt treatment (*N* = 153 individuals) before we ended the experiment when remaining live animals were manually removed from the egg sacs.

### Morphometric analysis

We found that newly hatched larvae in the 1,000 µS/cm conductivity level (across both salt types) were smaller on average than those in the other conductivity levels based on total length, snout-vent length (SVL), mid-section width, head width and tail width (*X*^*2*^_*1*_ = 6.52, *p* = 0.01; Table 3). The only significant difference in SVL was between the 1,000 µS/cm and the 300 µS/cm larvae (1.53 ± 0.54 mm, t.ratio = 2.9, *p* = 0.043; Table S2). While larvae from 100 to 600 µS/cm also had larger SVL’s than larvae in the 1000 µS/cm treatment, those differences were not significant (Table 3). Similar patterns were observed for mid-section width and head width, with the 1,000 µS/cm animals being slimmer (0.51 ± 0.14 mm, t.ratio = 3.63, *p* = 0.006) and having smaller head widths (0.65 ± 0.19 mm, t.ratio = 3.43, *p* = 0.011) than the animals in the 100 µS/cm treatment, and overall non-significantly smaller than both 300 and 600 µS/cm.

**Table 3:** Average larval morphometric measurements per conductivity level.

Conductivity Level	Total Length (mm)	SVL (mm)	Head Width (mm)	Midsection Width (mm)	Tail Width (mm)
100 µS/cm	46.44 ± 0.42	26.79 ± 0.23	7.66 ± 0.09	5.53 ± 0.08	2.84 ± 0.05
300 µS/cm	46.13 ± 0.33	26.95 ± 0.21	7.53 ± 0.06	5.39 ± 0.07	2.86 ± 0.05
600 µS/cm	46.45 ± 0.48	26.72 ± 0.31	7.44 ± 0.1	5.38 ± 0.09	2.83 ± 0.07
1000 µS/cm	44.67 ± 0.43	25.44 ± 0.27	7.04 ± 0.1	5.00 ± 0.09	2.72 ± 0.06
Mean	45.92 ± 0.42	26.48 ± 0.35	7.42 ± 0.13	5.32 ± 0.11	2.81 ± 0.03

### Ion concentrations in tank water

**Fig. 5:**
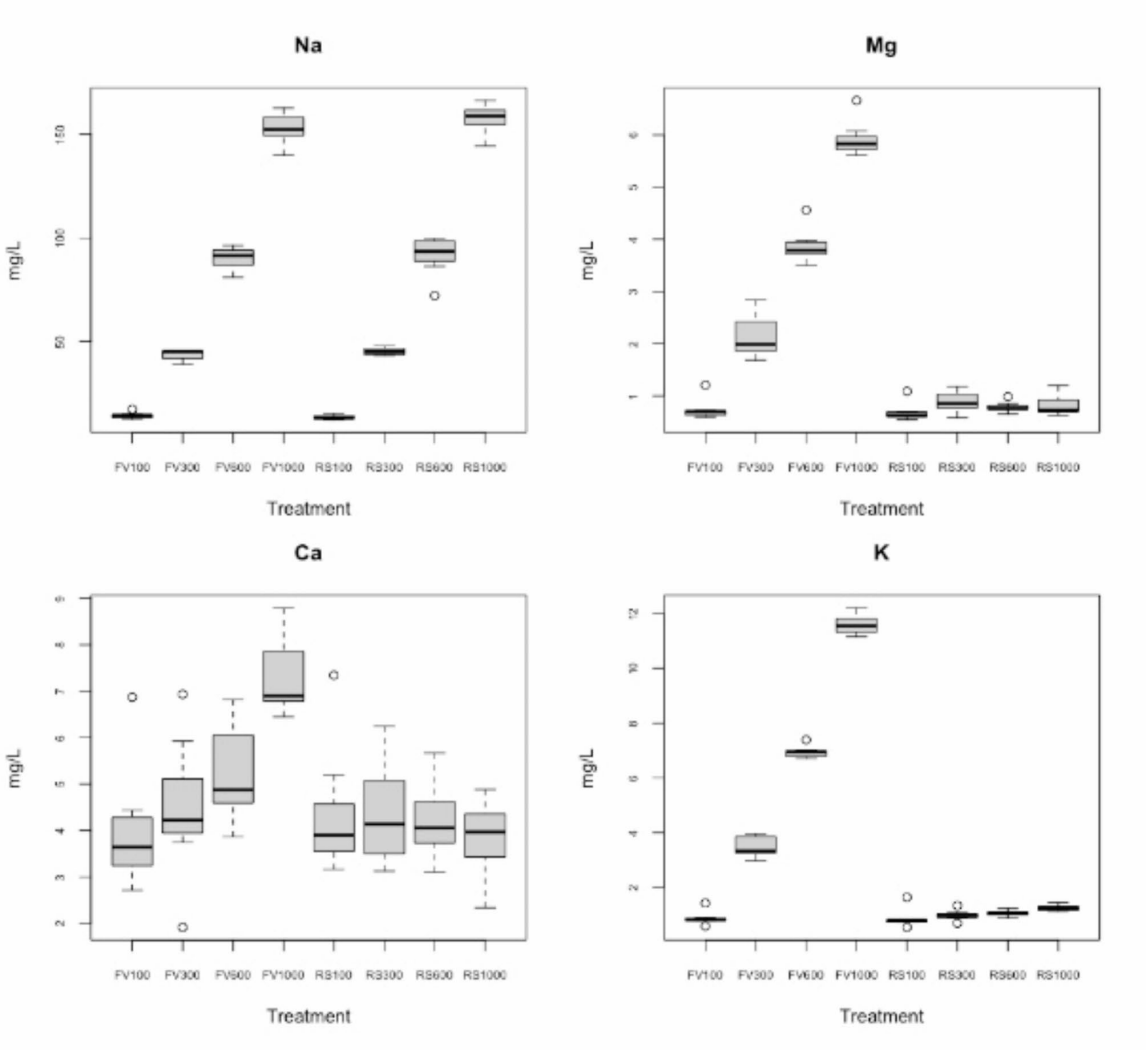
Boxplots of showing comparisons of ion concentrations for each salt treatment and conductivity level. Sodium was the most prevalent ion in both salt treatments and was found at similar concentrations; other ions were present at higher levels in the aquarium salt treatment. FV represents Flora Viv,* the aquarium salt product*,* and RS represents rock salt.*

Ion analysis showed nearly equal levels of sodium in both the aquarium salt and rock salt treatments (Fig. 5; ANOVA F = 0.891, p-value = 0.350 for the conductivity × salt treatment interaction). Sodium concentrations were highest in the 1,000 µS/cm level tanks (mean = 10 mg/L) and lowest in the 100 µS/cm (mean = 165 mg/L, Fig. 5). Concentrations of magnesium, calcium, and potassium were higher in the aquarium salt treatment, though at lower concentrations than sodium (< 13 mg/L) (ANOVA; conductivity × salt treatment interaction for the three ions: F = 1095.61, F = 35.31, F = 3964.82 for Mg^2+^, Ca^2+^ and K^+^, respectively; p-value < 0.0001 for all ions).

**Fig. 6:**
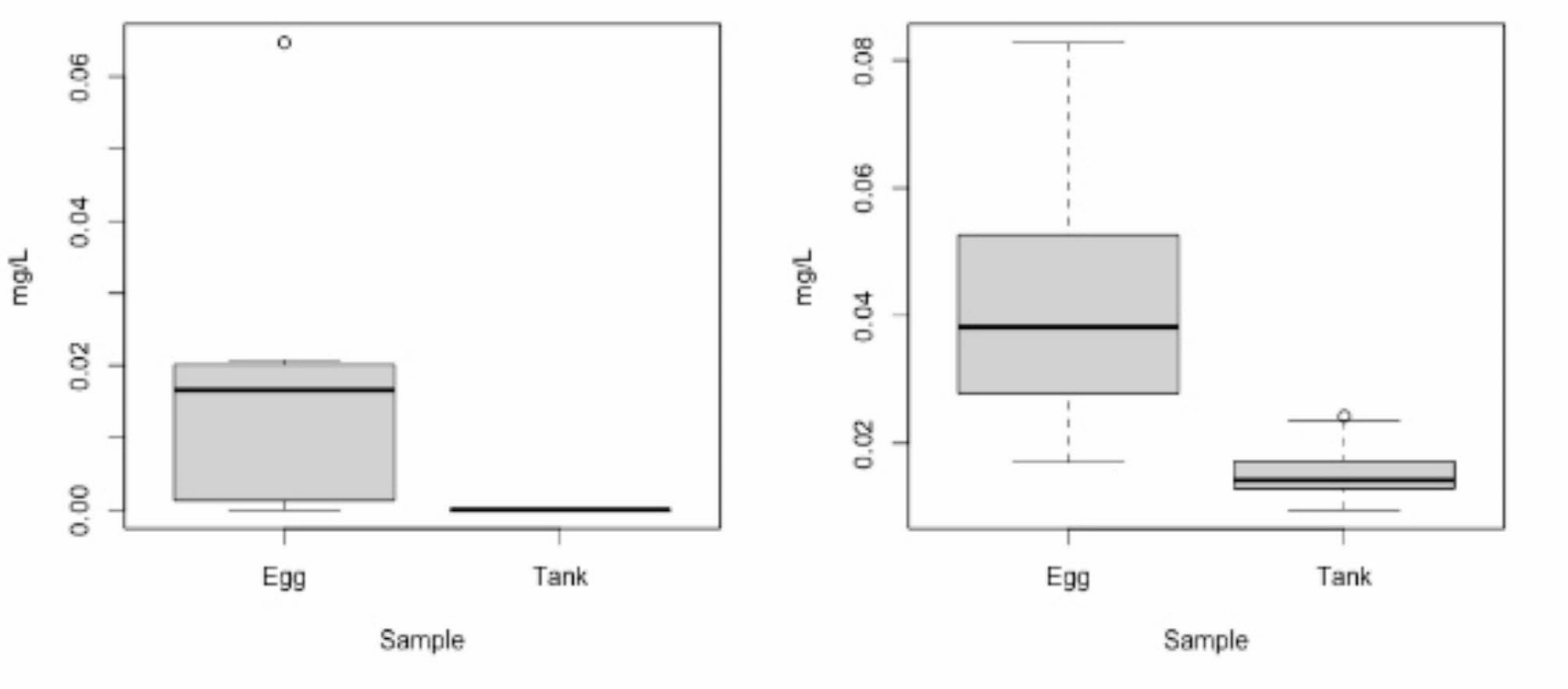
Comparison of (**a**) dissolved Fe and (**b**) dissolved Al ions present inside hellbender eggs vs. in tank water.

The inner egg samples showed the concentrations of most ions to be nearly equal to the surrounding water (Table S3), with the exception of Fe^2+^ and Al^3+^. Although occurring at low concentrations (< 0.1 mg/L), there was more Fe^2+^ (*X*^*2*^_*1*_ = 39.4, *p* < 0.001) and Al^3+^ (*X*^*2*^_*1*_ = 16.2, *p* < 0.001) in the inner egg fluid than there was in the tank water (Fig. 6).

## Discussion

We found that elevated conductivity does not result in high mortality of hellbender eggs and early-stage larvae, but does increase mortality after chronic exposure to high conductivities (> 600 µS/cm). While our counter-intuitive finding suggests that exposure to high conductivity does not lead to immediate mortality, we cannot rule out that there may be indirect, sublethal effects, either chemically, physiologically, or biologically that underlie the association between hellbender persistence in streams and increased conductivity. We expected the higher levels of conductivity (i.e., 600 and 1,000 µS/cm) to cause mortality in hellbender eggs and larvae, however our results showed that survival across both salt treatments and all conductivity levels was high (0.93). Although there was significantly higher mortality in individuals raised in the 1,000 µS/cm level than those raised in the 300 µS/cm level, survival across both salt treatments in the 1,000 µS/cm level was still > 0.85. Along with our results for high survival, our experiment indicates that conductivity may contribute to, but not be a sole factor for explaining the absence of hellbenders in streams with conductivity above certain thresholds (e.g., 278 µS/cm^26^) and that eggs and larvae potentially have a higher degree of tolerance to elevated water conductivity than previously reported. While hatching rates and survival estimates exist for wild nests in low conductivity situations^28^, we are unaware of such data for high (> 300 µS/cm) streams. Kaunert (2024)^28^ found that egg survival in nine attended nests in Pennsylvania streams with conductivity < 300 µS/cm (including locations for our egg collection), varied between 15% and 96%, but were frequently > 50%; larval survival to dispersal in three assessed nests was high (77–93%). Hopkins et al. (2023)^34^ evaluated the fate of multiple nests in Virginia and found frequent nest failure due cannibalism and relatively low larval survival, but did not report conductivity values, instead relying on watershed forest cover as a measure of stream and watershed quality.

Several other studies have found increased conductivity, influenced by NaCl specifically, to increase mortality in amphibians. Karraker et al. (2008)^35^ found overall lower survival in developing spotted salamander (*Ambystoma maculatum*) embryos when conductivity was raised to 500 µS/cm (mean = 0.68) and 3,000 µS/cm (mean = 0.03) compared to mean = 0.84 survival in control water with conductivities altered using rock salt. The same study found that while wood frog (*Lithobates sylvatica*) embryos were less sensitive to increased conductivity, they also had higher mortality as conductivity increased, with survival decreasing from mean = 0.91 to 0.77 to 0.41 in control, 500 µS/cm, and 3,000 µS/cm water respectively. We did not find differences in survival between the 100, 300, and 600 µS/cm treatments, suggesting that spotted salamander and wood frogs, both vernal pool breeding species, may be more sensitive to increased ion concentrations than hellbenders, though their highest conductivity tested (3,000 µS/cm) greatly exceeded our highest at 1,000 µS/cm. Green frogs (*Lithobates clamitans*), a pond and permanent wetland breeding species, are likely less sensitive than spotted salamanders, wood frogs, or hellbenders, and developing embryos have been found to have nearly 0.80 survival when conductivity was increased to 3,000 µS/cm, and > 0.93 survival in conductivities ≤ 1,500 µS/cm^36^. Lethal concentrations of NaCl, however, have been shown to be much higher for both green frog (8,140 µS/cm NaCl LC_50,96 h_, Jones et al., 2015), and wood frog tadpoles (5,230 µS/cm NaCl LC_50,96 h_^37^) than those tested in these experiments, which may indicate that while lower NaCl concentrations (< 3,000 µS/cm) do not cause immediate mortality, they may still have deleterious effects over time.

The higher mortality we observed at 1,000 µS/cm suggests that high concentrations of Na^+^ and Cl^−^ ions may induce stress in hellbenders, and there are still unknowns around possible sublethal effects of elevated conductivity. There are no natural aquatic systems however, where Na^+^ and Cl^−^ ions are not interacting with other ions present. For example, some regions within the hellbender distribution have dolomite and limestone bedrocks which are known to leach Ca^2+^, Mg^2+^, HCO_3_^−^, SO_4_^2−^, and Cl^−^, and conductivity is naturally higher in aquatic systems where these geologic formations are dominant^38^. In our system, the dominant rock types are sandstone and shale (Allegheny formation, Pennsylvanian; https://mrdata.usgs.gov/geology/state/sgmc2-unit.php?unit=PAPAa;6), which tend to have lower Ca^2+^, Mg^2+^ and Cl^−^ and higher Na^+^ concentrations due to weathering^39^. It is also widely known that higher Ca^2+^ concentrations in hard waters can significantly decrease toxicity of many metals to aquatic invertebrates and fish^40,41^. Soucek et al. (2011)^42^ demonstrated that toxicity of Cl^−^ from salinization in numerous aquatic invertebrates is significantly lower in hard water with high concentrations of CaCO_3_. Van Dam et al. (2010)^43^ reported lower tolerance and higher mortality of the freshwater snail *Amerianna cumingi* when exposed to a single type of salt (MgSO_4_) than when exposed to a combination of salts (magnesium and calcium) at a 9:1 proportion. While Gillis (2011)^44^ found that high Ca^2+^ provided similar protective effects in mussel glochidia exposed to high salinity water. Thus, conductivity as a sole measure of water quality for eastern hellbender may be misleading if not considered in tandem with water hardness and other stressors occurring simultaneously within watersheds (e.g., pesticide and fertilizer application, deforestation, mining, etc.), or if additive or synergistic interactions between various ions are ignored^45^.

An unexpected result was the delayed hatching time between the aquarium salt and rock salt treatments, with larvae in rock salt-based conductivities hatching 11 days earlier than in those in the aquarium salt treatments. An overall drawn-out time period of hatching was also an unexpected result with only 67% of larvae having hatched by the end of the experiment, which was ~ 2.5 weeks after hatching ended in the wild (M. Kaunert, pers. obs.). This experiment indicates that delayed egg hatching may be due to a more specific ionic difference between rock salt and aquarium salts. This mechanism for how specific ion composition, other than conductivity or NaCl, might influence the time to hatching of hellbenders, has potential effects on growth and survival of the young later in life. In hellbenders, it is unknown whether earlier hatching has any effects on larval fitness and survival, though it can be assumed that smaller, less-developed larvae are better protected from predation inside the egg sac. In our case, the rock salt treatment had slightly higher hatching success (77%) compared to aquarium salts (58%), and larvae began hatching 11 days earlier in the rock salt treatment, but there was little difference in survival and morphology between salt treatments. The delayed hatching rates across all treatments could be explained by variety of factors not included in our experiment, including no variation in temperature, lack of bacteria in the controlled tank water which may play a role in breaking down the egg membrane in the wild, or differences in agitation rates between our air stones and the behaviors of guardian males. Furthermore, mortality increased greatly in the final two weeks of the experiment, which may indicate that embryos had exhausted the time that being enclosed in eggs was beneficial, though nearly all of this mortality did occur in the higher conductivity levels (600 and 1,000 µS/cm, Fig. 3a).

A potentially deleterious effect of prolonged confinement within egg sacs was our finding of higher concentrations of Al^3+^ (0.017–0.083 mg/L) and Fe^2+^ (0–0.06 mg/L) in the egg sac fluid than the surrounding tank water (0.009–0.024 mg/L Al^3+^; ~ 0 mg/L Fe^2+^, Fig. 6). Although preliminary, the elevated aluminum and iron in samples of fluid from hellbender eggs suggested that a variety of metals or other pollutants might accumulate in higher concentrations inside eggs near developing embryos. This is potentially concerning given that dissolved aluminum is known to be harmful to aquatic biota including fish and amphibian eggs and larvae^46–49^. More work is needed to better understand the mechanism, but the accumulation of ambient contaminants in hellbender eggs could be of detriment to populations where higher levels of aluminum occur, particularly if these streams are also experiencing acidification.

The size difference between individuals raised in the 1,000 µS/cm and those raised in the lower conductivity levels suggest that high conductivity levels affect growth and development of larval hellbenders. Reduced size in amphibians is a well-documented plastic response to a variety of aquatic stressors, such as hydroperiod, temperature, water quality, competitor density, and predator density^20,50–52^. For example, Hopkins et al. (2013)^45^ found that rough-skinned newt (*Taricha granulosa*) embryos reared in increased concentrations of NaCl and MgCl_2_ resulted in larvae that were smaller, less developed, and hatched earlier than those reared in control conditions. Squires et al. (2010)^20^ found that brown treefrog (*Litoria ewingii*) larvae exposed to extremely high conductivities ranging from 9,000 to 24,700 µS/cm, grew slower and took longer to metamorphose than control larvae. When the same larvae were then returned to the control treatment, they compensated and grew at faster rates than the control larvae until there was no difference in size (~ 8 days). Smaller size at metamorphosis in amphibians has been associated with reduced fitness, lower foraging and predator avoidance ability, and higher mortality in later life stages^53–56^. For example, Székely et al. (2020)^55^ found that larger sized tadpoles of the pacific horned frog (*Ceratophrys stolzmanni*) had better locomotor performance and survival at time of metamorphosis, while Thompson and Popescu (2021)^56^ found that smaller wood frog metamorphs from fast drying ponds showed slower growth and development compared to control animals. Future studies that include longer periods of observation post-hatching may lead to more insights of effects on larval development, fitness, and foraging behavior once the larvae begin to feed.

Our findings indicate that stream conductivity should be taken into consideration for hellbender conservation, but that conductivity alone may be a less informative indicator of stream water quality for hellbenders than previously suggested. New research suggests that watershed-level forest cover can lead to higher levels of filial cannibalism (guardian males eating viable eggs)^34^. At the same time loss of forest cover was related to reduced recruitment and increased conductivity in Appalachian streams^8^. Thus, our study highlights an additional risk to hellbender populations from altered water quality and provides a critical link between experiments and field observations. Our study shows that specific ionic differences, rather than overall conductivity, can influence timing of hatching, and that higher conductivity can lead to higher mortality and smaller body size. It is possible that sublethal effects on size and hatching time found in our study are amplified by exposure to other stressors such as pollution, warmer temperatures, and sedimentation, which could further impair larval development and potentially lead to direct mortality and population suppression. In addition, variation in conductivity and ion load levels during the embryonic and larval development could alter the effects on size and survival. Kaunert (2024)^28^ found that conductivity levels in active nests in Pennsylvania streams fluctuated between September and November, but the fluctuations were low compared to the range of exposure in our experiment: mean ± SD = 70.17 ± 11.50 µS/cm (for *n* = 10 measurements) for the nest with the lowest overall conductivity and 307.56 ± 20.78 µS/cm (for *n* = 13 measurements) for the nest with the highest overall conductivity). Future experiments with longer exposures to specific profiles of ions post-hatching could provide critical insights into the mechanism of altered hatching time and success, and whether the differences in survival between the low and high conductivity levels in this study increase or remain constant through time. Common garden-type experiments with eggs from streams with low and high conductivity levels could also elucidate the potential for selection of increased tolerance to high conductivity levels. In addition, additional growth or locomotor performance trials could evaluate whether the differences in body size observed in our study are biologically relevant.

This work also has implications for conservation management strategies such as reintroductions, translocations, and watershed management. Management agencies are undertaking efforts to reintroduce hellbenders in streams within their historical range or augment existing populations through head-starting efforts. However, releasing animals into high conductivity streams may still hinder population augmentation, leading to suboptimal conservation efforts. As such, given the unknowns around the synergy of elevated conductivity with other stressors, reintroductions should target streams with low conductivities (e.g., < 300 µS/cm); otherwise, in the absence of water quality improvement efforts, reintroductions could be less successful in the long term. Overall, our results support other studies calling for a multi-prong approach to eastern hellbender conservation, including investigations of water chemistry and additional experimental research on early life stages of one of North America’s most iconic and imperiled amphibians, the snot otter.

## Electronic supplementary material

Below is the link to the electronic supplementary material.

Supplementary Material 1

## Data Availability

Data are available from the corresponding authors, R. K. Brown and V. D. Popescu, upon request.
