# Supplementary material for: Assessing the effects of conductivity on egg development and survival of Eastern Hellbenders (Cryptobranchus a. alleganiensis)
Source: Sci Rep. 2025 Feb 25;15:6821. doi: 10.1038/s41598-024-82969-5 (PMC11861594; doi:10.1038/s41598-024-82969-5)
Supplement: Supplementary file 1 — Supplementary Material 1 [file 41598_2024_82969_MOESM1_ESM.docx]

# Assessing the Effects of Conductivity on Egg Development and Survival of Eastern Hellbenders (*Cryptobranchus a. alleganiensis*)

**Supplementary Material**

**Table S1.** *Pairwise comparison of mortality rate by conductivity level* *showing significantly more mortality in the 1,000 µS/cm treatment than the 300 µS/cm treatment with significant values in bold.*

| **contrast** | **estimate** | **SE** | **z.ratio** | **p.value** |
| --- | --- | --- | --- | --- |
| 100-300 | -0.416 | 0.923 | -0.451 | 0.6522 |
| 100-600 | 1.036 | 0.692 | 1.497 | 0.4035 |
| 100-1000 | 1.666 | 0.653 | 2.55 | 0.0539 |
| 300-600 | 1.452 | 0.803 | 1.808 | 0.2826 |
| 300-1000 | 2.082 | 0.77 | 2.704 | **0.0411** |
| 600-1000 | 0.63 | 0.468 | 1.345 | 0.4035 |

| **Table S2.** *Pairwise comparisons of hellbender larvae SVL by conductivity level with significant values in bold.*  **Pairwise comparisons (SVL)** | | | | | |
| --- | --- | --- | --- | --- | --- |
| **Contrast (**µS/cm) | **estimate** | **SE** | **df** | **t.ratio** | **p.value** |
| 100-300 | -0.2764 | 0.531 | 33.4 | -0.52 | 1.000 |
| 100-600 | -0.0109 | 0.53 | 34.5 | -0.021 | 1.000 |
| 100-1000 | 1.2553 | 0.549 | 31 | 2.288 | 0.119 |
| 300-600 | 0.2654 | 0.484 | 60.2 | 0.549 | 1.000 |
| 300-1000 | 1.5317 | 0.537 | 34.7 | 2.852 | **0.044** |
| 600-1000 | 1.2663 | 0.536 | 36 | 2.361 | 0.119 |

**Table S3.** *Total composition of measured ions in the 8 inner egg fluid samples (mg/L).*

| **Ion** | **100 Aq. Salt** | **100 Rock Salt** | **300 Aq. Salt** | **300 Rock Salt** | **600 Aq. Salt** | **600 Rock Salt** | **1000 Aq. Salt** | **1000 Rock Salt** |
| --- | --- | --- | --- | --- | --- | --- | --- | --- |
| **Al** | 0.053 | 0.036 | 0.039 | 0.083 | 0.052 | 0.038 | 0.019 | 0.017 |
| **Ca** | 6.871 | 7.344 | 6.934 | 6.246 | 6.541 | 5.670 | 8.632 | 4.883 |
| **Fe** | 0.019 | 0.018 | 0.023 | 0.065 | 0.000 | 0.016 | 0.000 | 0.000 |
| **K** | 1.430 | 1.643 | 3.790 | 1.342 | 7.390 | 1.233 | 12.197 | 1.364 |
| **Mg** | 1.206 | 1.087 | 2.838 | 1.177 | 4.560 | 0.985 | 6.660 | 0.991 |
| **Na** | 15.030 | 15.189 | 45.510 | 43.525 | 96.260 | 98.587 | 152.349 | 159.413 |
